# Autologous cell-based therapy for treatment of large bone defects: from bench to bedside

**DOI:** 10.1007/s00068-018-0906-y

**Published:** 2018-01-19

**Authors:** R. Verboket, M. Leiblein, C. Seebach, C. Nau, M. Janko, M. Bellen, H. Bönig, D. Henrich, I. Marzi

**Affiliations:** 1 Department of Trauma-, Hand- and Reconstructive Surgery, University Hospital Frankfurt, Frankfurt, Germany; 2 Department of Transfusion Medicine and Immune Hematology, University Hospital Frankfurt and DRK Blood Donor Service Baden-Württemberg-Hessen, Frankfurt, Germany

**Keywords:** Bone defect, Cell therapy, Stem cells, BMC, Bone marrow mononuclear cells, Regeneration

## Abstract

**Objectives:**

Reconstruction of long segmental bone defects is demanding for patients and surgeons, and associated with long-term treatment periods and substantial complication rates in addition to high costs. While defects up to 4–5 cm length might be filled up with autologous bone graft, heterologous bone from cadavers, or artificial bone graft substitutes, current options to reconstruct bone defects greater than 5 cm consist of either vascularized free bone transfers, the Masquelet technique or the Ilizarov distraction osteogenesis. Alternatively, autologous cell transplantation is an encouraging treatment option for large bone defects as it eliminates problems such as limited autologous bone availability, allogenic bone immunogenicity, and donor-site morbidity, and might be used for stabilizing loose alloplastic implants.

**Methods:**

The authors show different cell therapies without expansion in culture, with ex vivo expansion and cell therapy in local bone defects, bone healing and osteonecrosis. Different kinds of cells and scaffolds investigated in our group as well as in vivo transfer studies and BMC used in clinical phase I and IIa clinical trials of our group are shown.

**Results:**

Our research history demonstrated the great potential of various stem cell species to support bone defect healing. It was clearly shown that the combination of different cell types is superior to approaches using single cell types. We further demonstrate that it is feasible to translate preclinically developed protocols from in vitro to in vivo experiments and follow positive convincing results into a clinical setting to use autologous stem cells to support bone healing.

## Background

Reconstruction of long segmental bone defects is demanding for patients and surgeons, and associated with long-term treatment periods and substantial complication rates in addition to high costs. Large bone defects result from major trauma, surgical excision of tumors, debridement after posttraumatic septic non-unions, osteitis or explantation of endoprothesis. While defects up to 4–5 cm length might be filled up with autologous bone graft, heterologous bone from cadavers, or artificial bone graft substitutes, current options to reconstruct bone defects greater than 5 cm consist of either vascularized free bone transfers, the Masquelet technique or the Ilizarov distraction osteogenesis [1–3].

Alternatively, autologous cell transplantation is an encouraging treatment option for large bone defects as it eliminates problems such as limited autologous bone availability, allogenic bone immunogenicity, and donor-site morbidity, and might be used for stabilizing loose alloplastic implants [4, 5]. Until now, systematic clinical studies applying autologous bone cell transplantation have barely performed.

In contrast to the extensive in vitro and animal experiment data, there are only few studies that show clinical results for cell therapy treatments to regenerate bone.

There are two clinical application forms of cell therapies to regenerate bone. Besides the biological differences, various health law-related consequences also emerge for the manufacturer and the orthopedic surgeon in attendance.

1. Cell therapies without expansion in culture
2. Cell therapies with ex vivo expansion

### Cell therapies without expansion in culture

Cells are harvested or produced during an operation. Bone marrow aspiration concentrate (BMAC) is a typical example of this form of application. At the beginning of the operation, a defined volume of bone marrow is harvested by vacuum aspiration of the ventral or dorsal iliac crest and suspended in an anti-coagulating heparin and anticoagulant citrate dextrose solution in a transfusion bag. Mononuclear cells are then isolated from the harvested bone marrow aspirate in a density gradient centrifuge in the closed system that has been used since 2005.

In a prospective clinical study and in various experimental treatments, the research group of Jäger et al., has successfully treated over 100 patients with local bone-healing disorders using a BMAC biomaterial composite [6]. Fifty percent of the bone defects were grafted with autologous cancellous bone and the remaining 50% with a BMAC biomaterial composite (hydroxylapatite, Orthoss®, Geistlich, Wolhusen, Switzerland versus collagen sponge, Gelaspon®, Chauvin Ankerpharm, Berlin, Germany). So far, the study has found that the use of BMAC reduces the harvest of autogenous bone by 50% with no slowing down or absence of bone healing being observed [7, 8]. No complications with the application were observed in any of the patients. The low complication risk of this procedure [9] and the osteogenic potency in the parallel application of different biomaterials has also been reported by other research groups [10, 11]. However, a confirmatory clinical study under the current regulatory requirements has not been reported to our knowledge.

### Cell therapies with ex vivo expansion

In orthopedics and traumatology, autologous cell therapies have been used regularly on the musculoskeletal system after ex vivo cultivation, at least since the clinical introduction of autologous chondrocyte transplantation (ACI). Unlike cartilage regeneration, for which ACI was used in more than 12,000 patients between 1987 and 2005, [12, 13] there are no reliable data on osseous regeneration after temporary in vitro cultivation. In the treatment of necrosis of the femoral head, for instance, whereas numerous one-step transplantations are documented, only three case studies with a maximal observation period of 3 months can be found. Here, a mixed cell population from bone marrow cells (so-called tissue repair cells, TRCs) was expanded over 12 days under GMP conditions and then transplanted autologously together with a scaffold made of tricalcium phosphate (TCP) within the framework of core decompression [14].

A new started study with ex vivo expanded mesenchymal stromal cells (hBM- MSCs) is the Orthounion study. This study is a multi-centre, open, comparative, three-arm, randomized clinical trial (EudraCT-No. 2015-000431-32) to compare the efficacy of autologous human bone marrow-derived expanded mesenchymal stromal cells treatments versus iliac crest autografts. Bone healing in patients with diaphyseal and/or metaphysodiaphyseal fractures, atrophic or oligotrophic non-union is investigated. The project started on 1st January 2017.

The particular drawbacks of temporary cultivation of MSCs lie not only in the considerable logistical effort to ensure the quality of the cell therapy treatment but especially in the biological characteristics of this cell population. As soon as MSCs are isolated from their tissue mass and transferred to a culture dish, differentiation proceeds in accordance with the culture conditions [15–17]. The yet inconclusive biological effects when fetal bovine serum is used in the culture, as well as telomere shortening, and thus cell aging with ex vivo cultivation also have to be considered. Furthermore, analysis of 170 neoplasia-associated DNA promoters was able to show that despite the relatively high genetic stability of MSCs from human bone marrow or adipose tissue, damage in the genome could occur at later stages [18]. The question as to whether these genotoxic effects of prolonged in vitro cultivation are also clinically manifested after re-transplantation remains unanswered, however. The potential effects of changes in the chromatin structure due to epigenetic factors at the beginning of osteoblastic differentiation also remain largely unknown [19].

### Cell therapy in local bone defects, bone healing disorders and osteonecrosis

Other research groups have also reported positive clinical results after using human bone marrow cells. Giannini et al. showed that in patients with osteochondral defects in the talus, functional improvements were achieved through autologous bone marrow cell transplantation by arthroscopic surgery [20]. As early as 1991, Conolly et al. [21] reported equivalent healing rates for autologous bone marrow grafting to treat post-traumatic pseudarthrosis of the tibia. Other authors also support the high osseous regeneration potency of the percutaneous implantation of autologous bone marrow concentrate to treat pseudarthrosis [22, 23] and discuss supplementary osteoblastic stimulation using platelet-rich plasma (PRP) [21].

Although the underlying mechanism for the regeneration process is not completely understood, essentials constitutes have been assumed besides biomechanical stability and vascularization in accelerating new bone formation: growth factors, osteoprogenitor cells and extracellular matrix/natural scaffolds.

An overview about published studies after cell therapy in bone defects or bone healing disorders are summarized in Table 1.

**Table 1:** Overview about published studies after cell therapy in bone defects or bone healing disorders

Author	Year	Journal	Bone defect	*N* patients	Results
Connolly et al. [21]	1991	CORR	Pseudarthrosis	20	Application of autologous bone marrow (BM) in tibial pseudarthrosis or “non-union”. Post-operative treatment with plaster cast. Additional intramedullary nailing in ten cases. The authors report that autologous BM application produced the same results as for autologous bone transplantation
Lokiec et al. [76]	1996	JBJS-Br	Simple	10 bone cysts	Percutaneous injection of autogenous bone marrow: all the patients became pain-free after 2 weeks and resumed full activities within 6 weeks. The cysts were radiologically consolidated and showed remarkable remodeling within 4 months. Bone healing was achieved 12–48 months after treatment (no complications)
Köse et al. [77]	1999	Bull Hosp J T Dis	Simple	12 bone cysts	Autologous bone marrow injection in bone cysts: complete healing occurred in two patients, whereas three cysts showed residual defects. In six patients, cysts recurred. Authors concluded that factors such as the size, multi-loculation, and completeness of the filling of the cyst with bone marrow grafting might influence the post-operative outcome
Hernigou et al. [78]	2002	CORR	AVN (Hip)	116 (189 hips)	Evaluation of the clinical outcome 5–10 years after core decompression in combination with injection of autologous BM concentrate in the treatment AVN of the femoral head. Very good results in pre-collapse stages (ARCO I-II): 9 out of 145 hips were replaced endoprosthetically. In post-collapse stages, 25 out of 44 hips replaced endoprosthetically. Better results with higher CFU-F and cell numbers
Rougraff et al. [79]	2002	JBJS-Am	Unicameral	23 bone cyst	Percutaneous injection of allogeneic demineralized bone matrix augmented with autogenous bone marrow is an effective treatment for unicameral bone cysts
Chang et al. [80]	2002	JBJS-Br	Unicameral bone cyst	79	14 patients treated with BM (27 injections) vs. 65 patients with steroid application (99 injections). Repeated injections were required in 57% of patients after BM had been used and in 49% after steroid. No complications. No advantage could be shown for the use of autogenous injection of BM compared with injection of steroid in the management of unicameral bone cysts
Price et al. [81]	2003	Spine	Spinal fusion	77	Retrospective study with three different bone grafting techniques: autologous iliac crest bone graft (ICBG) vs. freeze-dried corticocancellous allograft vs. composite graft of autologous bone marrow (BM) and demineralized bone matrix. Segmental instrumentation with dual-rod fixation was used in 77 patients. No BM aspiration-associated morbidity. Fusion rates were comparable for ICBG and BM group
Docquier et al. [82]	2003	J Pediatr Orthop	Simple bone cyst	17	Percutaneous aspiration and injection of BM. FU: 33.9 months. Slow regression of the cyst and progressive healing: 13 cases (76%). No response: 2 cases (12%), recurrence: 2 cases (12%)
Gangji et al. [83]	2004	JBJS-Am	AVN (hip)	13 (18 hips)	Necrosis of the femoral head in ARCO stages I-II. Core decompression (vs. core decompression + BM aspirate (ten patients). Within 24 months, significant reduction in pain, functional improvement and lower AVN progression rate after cell therapy. No transplantation-related complications
Hernigou et al. [84]	2005	JBJS-Am	Pseudarthrosis/non-unions (atrophic, tibia)	60	Injection of 20 cm^3^ BM concentrate: 612 ± 34 progenitor cells/cm^3^ in the aspirate compared to 2579 ± 1121 progenitor cells/cm^3^ after density gradient centrifugation: healing in 53 cases. Positive correlation between callus regeneration and the number of CFUs
Kanellopoulos et al. [85]	2005	J Pediatric Orthop	Active unicameral bone cyst	19	BM injection in bone cysts. All patients were asymptomatic at the latest follow up. Two patients required a second intervention to achieve complete cyst healing. Radiographic outcome was improved in all patients according to the Neer classification at the latest FU. There were no significant complications related to the procedure, nor did any fracture occur after initiation of the above regimen
Neen et al. [86]	2006	Spine	Spinal fusions	50	Therapy using HA-collagen I composite incubated with autologous BM aspirate (incubation time: 20 min) vs. autologous bone transplantation. The same posterolateral lumbar fusion rates for both groups, similar functional results for both groups. Autologous bone transplantations raised the fusion rate in “interbody fusions”, but donor-site morbidity in 14% of the cases
Yan et al. [87]	2006	Chin J Traumatol	AVN (hip)	28 (44 hips)	Percutaneous multiple hole decompression combined with autologous BMCs. The earlier the stage, the better the result. A randomized prospective study needed in the future to compare with routine core decompression
Deng et al. [88]	2007	Chin J Regen Reconstr Surg	Bone cyst	13	Transplantation of the autologous bone marrow combined with the allograft bone. Complete healing within 3.5–8 months (Ø 5.2 months). No recurrence, no pathological fracture occurred. Complete recovery of function
Cho et al. [89]	2007	JBJS-Br	Bone cysts	28 (58)	30 patients treated by steroid injection vs. 28 individuals by bone marrow grafting. Overall success rates: 86.7 vs. 92.0%, respectively (*P* > 0.05). Initial success rate: 23.3% in the steroid group vs. 52.0% in the BM group. Mean number of procedures: 2.19 (1–5) vs. 1.57 (1–3) (*P* < 0.05). Average healing interval: 12.5 months (4–32) *P* = 14.3 months (7–36) (*P* > 0.05). Rate of recurrence after initial procedure: 41.7 vs. 13.3% (*P* < 0.05). Although the overall rates of success of both methods were similar, the steroid group showed higher recurrences after a single procedure and required more injections to achieve healing
Wright et al. [90]	2008	JBJS-Am	Bone cysts	77	Randomized, prospective study. Two therapy groups: injection of autologous BM (A) vs. injection of methylprednisolone (B). Healing rate within two years: 23% (A) vs. 42% (B). No significant difference in the functional outcome
Park et al. [91]	2008	Foot Ankle	Bone cysts	20 (23 cysts)	Therapy of unicameral bone cysts of the calcaneus. Two therapy groups: open surgery application of avital allogenic donor bone + autologous BM (A) vs. injection of demineralized bone powder + autologous BM (B). Healing rate within 49.4 months: A: 9 out of 13 cysts vs. B: 5 out of 10 cysts. No infections
Gan et al. [92]	2008	Biomaterials	Spinal fusions	41	Application of TCP incubated with BM concentrate (duration circa 2 h). Concentration factor (CFUs-ALP: 4.3). Drop in MSCs with increasing age, but no dependency on gender. After 34.5 months, spinal fusion in 95.1% of the cases
Zamzam et al. [93]	2008	Int Orthop	Solitary bone cysts	28	A minimum one-off percutaneous injection of autologous BM. No complications. Within 34.7 ± 6.87 months, bone healing in 82% of the cases
Jäger et al. [6]	2009	CSCRT	Bone defects	10	Significant bone regeneration through bone marrow concentrate (BMAC) in combination with autologous cancellous bone
Hendrich et al. [9]	2009	Orthop Rev	Bone defects, AVN	101	Proof of the low complication risk of autologous BMAC in 101 applications
Giannini et al. [20]	2009	CORR	Osteochondral lesions (talus)	48	Functional improvements after arthroscopy-assisted application of autologous BM aspirate in osteochondral defects in the talus
Sir et al. [94]	2009	Vnitr Lek	Fracture-related bone defects, pseudarthrosis	11	Local and one-step injection of MSCs from human BM. Results pending
Kitoh et al. [95]	2009	J Pediatr Orthop	Tibial vs. femoral lengthening osteotomies	28 (51 osteotomies)	Retrospective study. Application of ex vivo cultivated MSCs together with PRP Control group: 60 patients without MSC/PRP. No stimulation of bone healing by MSC/PRP. Worse results for the tibia
Hernigou et al. [96]	2009	Indian J Orthop	AVN (hip)	342 (534 hips)	Autologous cell therapy in ARCO stages I–II in combination with a core decompression. After 8–18 years, 94 endoprosthetic hip replacements. Predictor for a therapy success was a high number of progenitor cells
Wang et al. [97]	2009	Arch Orthop Trauma Surg	AVN (hip)	45 (59 hips)	BMAC injection in AVN of the femoral head (ARCO stage I–III). Clinically successful in 79.7%. Hip replacement within FU in 11.9% of the hips. Radiologically, 14 of the 59 hips exhibited femoral head collapse or narrowing of the joint space. Overall failure rate: 23.7%. The concentration factor of mononuclear cells from BM vs. BMAC was about 3
Miller et al. [98]	2010	Int Orthop	Non-union or segmental defect	13	Bone marrow cells harvested by a reamer-irrigator-aspirator (RIA) were treated by dexamethason and transplanted into segmental bone defects. Promising results were achieved using this technique; and given the complexity of these cases, the observed success is of great value and warrants controlled study into both standardization of the procedure and concentration of the grafting material
Yamasaki et al. [99]	2010	JBJS-Br	AVN (hip)	22 (30 hips)	Transplantation of bone-marrow-derived mononuclear cells (BMMNCs) combined with hydroxypapatite (HA) vs. HA only in AVN of the femoral head. Reduction of the osteonecrotic lesion was observed subsequent to hypertrophy of the bone in the transition zone in the BM group. In 3 patients of the BMMNC group, progression to extensive collapse occurred. Control group showed bone hypertrophy, but severe collapse of the femoral head occurred in 6 of 8 hips
Gessmann et al. [100]	2012	Orthop.Rev	Posttraumatic bone defect	8	Bone marrow aspiration concentrate (BMAC) was percutaneously injected in the centre of the regenerate at the end of the distraction phase by using a modified Ilizarov external frame using an intramedullary cable transportation system. Bony consolidation of the regenerate was achieved in all eight cases. No adverse effects of cell injection into the regenerate was seen
Kassem et al. [101]	2013	Acta Orthop Belg	Delayed union or non-union	20	Patients with internally fixed fractures with delayed union or non-union were treated with a bone marrow injection. The bone marrow aspirate was injected percutaneously into the fracture site. Nineteen out of the twenty fractures achieved clinical and radiological union, the injection appeared as a simple and effective method to accelerate fracture healing
Lee et al. [102]	2014	Clin Orthop Relat Res	Distraction osteogenesis (tibia)	22	Autologous BMAC were combined with PRP injection at the osteotomy site in distraction osteogenesis of the tibia. The treatment group showed faster healing at each cortex and full weight bearing was permitted earlier in the treatment group than in the control group, although the effect size was small
Desai et al. [103]	2015	HSS J	Delayed union or non-union	49	Percutaneous BMAC injection was combined with either DBM and/or rhBMP-2 in delayed union or non-union patients. It was shown to be a safe and effective treatment regardless of the fracture gap size or fracture site
Hernigou et al. [104]	2015	Int Orthop	Non-union (ankle)	86	Diabetic patients were treated with bone marrow mesenchymal stem cells (BM- MSCs) delivered in an autologous bone marrow concentrate (BMC). Treatment with BMC promoted non-union healing in 70 among 86 diabetic patients with a low number of complications. Treatment with BM-MSCs showed improved healing rates compared with standard iliac bone autograft treatment

The substantial requirements of bone healing are summarized by Giannoudis et al. in the diamond concept of bone fracture healing. That concept considered the mechanical environment, osteogenic cells, vascularity, osteoconductive scaffolds and growth factors [24] as essential factors for successful tissue engineering-based bone healing approaches. In line with that concept, Drosse et al. discussed a multi-component approach for tissue engineering of bone defects ranging from cell-based to scaffold-based approaches also including the use of osteogenic growth factors and genetic engineering [25].

As a basis for bone healing, therefore, mechanical stability, osteoconductive scaffolds, and a sufficient vascular bed are the basis for bone healing [24]. The role of growth factors is important as well, but one can assume that viable cells and vascularization allow the secretion of relevant factors, but it is unclear if this is sufficient. Better results in healing have been shown by application of growth factors [26, 27]. Thus, the addition of stem cells to a bone defect filled up with scaffolds in a vascularized environment appears to be a prospective, but challenging aim.

**Fig. 1:**
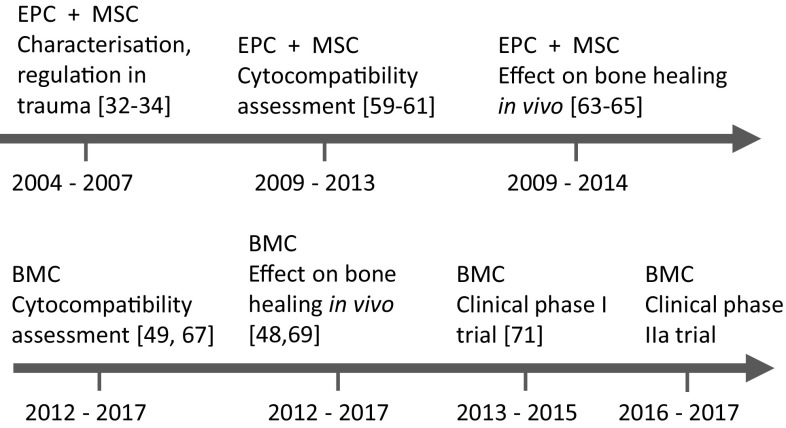
Timeline of our research efforts in the area of (stem)cells to understand and develop a clinically applicable protocol for a (stem)cell-based therapy of large bone defects

Considering these aspects, we started to investigate essential components obligatory for bone tissue engineering to develop a clinically applicable protocol for (stem)cell-based treatment of bone defects. The aim of this review is to trace back our research in this field from initial experiments to isolate and characterize stem cell populations, to evaluate suitable biomaterials in vitro, to proof the effect of regenerative cells and biomaterials in vivo, and based on that, to apply for and conduct first clinical trials to assess safety, feasibility (phase I) and the effect (phase IIa) of autologous bone marrow mononuclear cells (BMC) transplanted into the defect site on the bone healing (Fig. 1). All over, it took about 10 years to come from cell culture into humans and clinical trials are now ongoing.

### Cells

We followed the hypothesis that the combination of cells with complement properties might be more effective for the bone defect healing compared to approaches using single cell sorts. Endothelial progenitor cells (EPC) as potentially proangiogenic acting cells as well as marrow stromal cells (MSC) capable of forming new bone tissue were selected. A short overview on the biology of both cell types will be provided in the following. In this regard bone marrow mononuclear cells (BMC), as being a mixture of different cell types, are also worth to be mentioned.

#### Endothelial progenitor cells (EPC)

At least two major types of endothelial cell lines can be obtained by in vitro culture of mononuclear cells; first, the so-called “endothelial-like cells” or “early EPC” and second, the so-called “outgrowth EPC” or “late EPC”. Early EPC were used in all of our experimental projects. These cells are supposably derived from monocytic/dendritic cells co-expressing some endothelial markers together with leukocyte markers and demonstrating a high VEGF synthesis, some investigators hence designate them as endothelial-like differentiated PBMC [28, 29]. In the following ‘early EPC’ will be referred to as EPC. These cells can be generated in a sufficient amount within 3–5 days from a tenable volume of blood [30]. The contribution of early EPC in forming blood vessels is a matter of debate. Crosby and colleagues have reported that 8.3–11.2% of endothelial cells which developed in sponge-induced granulation tissue over 1 month were derived from circulating hematopoietic progenitor cells [31]. So it has been proposed that early EPC more likely act in a paracrine manner, secreting proangiogenic factors such as VEGF [28]. Own previous work indicated that early EPC are activated after multiple trauma by increased VEGF and TGF-β [32] but are harmed by increased concentrations of TNF-α, IL-1β [33] and activated neutrophils [34].

In contrast, outgrowth EPC or late EPC are characterized by a broad spectrum of endothelial markers including VEGF-R2 and UEA-I-Lectin. They express CD34, lack myeloid markers (CD45) and can be expanded in vitro. It is likely that these cells are generated from bone marrow-derived CD133 + cells [35, 36]. The culture period of late EPC is much longer, compared to that of early EPC. Single colonies of late EPC appear after 3–4 weeks [37], whereas early EPC require only 3–5 days [38, 39]. We observed that late EPC were also activated by musculoskeletal trauma [40], and that migration of late EPC towards injured tissue is impaired in elderly patients probably due to a reduced capability for VEGF synthesis [41].

#### Marrow stromal cells (MSC)

MSC were primarily described by Friedenstein et al. [42] as plastic-adherent cells or colony forming unit fibroblasts based on their adherence to tissue culture surfaces. MSC own a high proliferative potential and are phenotypically characterized by surface expression of CD71, CD73, CD90 and CD105, and the absence of the leukocyte marker CD45 or markers expressed by hematopoietic stem cells such as CD34 [43]. These cells can be functionally characterized by their potential for trilineage differentiation towards the adipogenic, the chondrogenic or the osteogenetic lineage in dependency from the presence of specific substances in the culture medium. For bone tissue regeneration, MSC combined with an appropriate scaffold have shown to support bone repair [43, 44].

We observed an increased proliferative activity of MSC in patients with multiple trauma and a decreased concentration in the bone marrow of patients who developed an atrophic non-union during our initial research [45].

#### Bone marrow mononuclear cells (BMC)

**Fig. 2:**
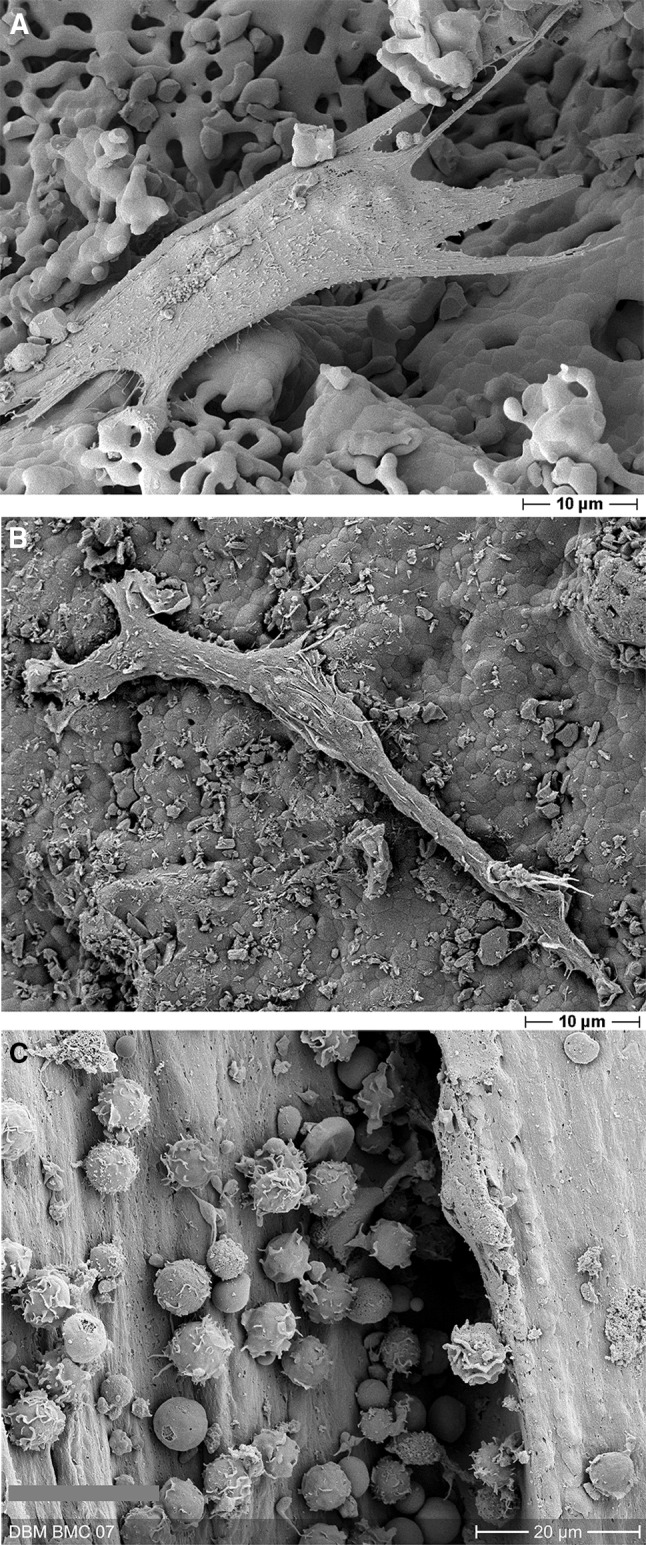
Cells with regenerative potential used in our research, SEM images. MSC (**a**), EPC (**b**) and BMC (**c**) on various types of scaffold (A: β-TCP; B: β-TCP; C: demineralized bone matrix) 2 days after seeding in vitro. Cells were fixed with glutardialdehyde, dehydrated and finally treated with dihydroxydisilazane overnight before being sputtered with gold

BMC are a heterogeneous mixture of diverse cell types containing (immature) lymphocytes, (immature) monocytes and progenitor cell populations. A BMC preparation evidentially comprises several subsets of regenerative potential such as (immature) monocytes and hematopoietic stem cells (HSC), a putative source of EPC, and precursors of MSC [46–49]. Putative MSC precursors can be identified by the expression of the nervous growth factor receptor-1 (CD271) and the absence of the pan leukocyte marker CD45 [50], whereas EPC can develop from CD34/CD133/CD45 expressing cells [51]. MSC precursors are a rare population of cells residing in the bone marrow that were defined by the presence of CD271 expression and, respectively, low or absence of pan leukocyte antigen CD45 expression. Those cells were frequently found in close proximity to CD34 + progenitor cells [50] and possess the potential for trilineage differentiation (adipogenic, chondrogenic, osteogenic potential) [52]. It has been shown furthermore that the CFU-F concentration correlates well with the concentration of those cells within the bone marrow [53] and that approximately 5% of those cells were capable to form CFU-F [50, 53]. It has been demonstrated that BMC support therapeutic effects by improvement of vascularization as exemplarily demonstrated by Jeon et al. [54] using the hind limb ischemia model of the mouse. Transplantation of BMC resulted in significantly increased microvessel density [54]. Interestingly, in cardio-vascular cell transplantation studies, BMCs were mostly applied in large successful studies (Assmus et al.) [46]. BMCs are easily taken by bone marrow aspiration of the iliac crest and processed for further clinical use. The different cells with regenerative potential are shown in Fig. 2.

#### Cells sources

Stem cells for bone tissue engineering can be harvested from different sources. Human MSC and EPC can be obtained not only from iliac crest bone marrow but can also be isolated from marrow of the femur using a Reamer Irrigator Aspirator (RIA). The application of a Reamer/Irrigator/Aspirator (RIA) system allows the harvest of vital bone marrow from the femur by continuous irrigation and simultaneous aspiration of the irrigation fluid. The irrigation fluid as well as the osseus particles within the irrigation fluid can be harvested using a filter. Actual studies demonstrate that the reaming debris obtained with RIA contains elevated levels of FGF-1, PDGF, IGF-1, TGF-β1, and BMP-1 in comparison with samples obtained from the iliac crest using needle puncture/aspirate technique [55]. Moreover, it was reported recently that human reaming debris is a rich source of multipotent stem cells. The harvested cells exhibit a phenotype and a plasticity commonly attributed to MSC in culture [56]. Own work has shown that in comparison with aspirates obtained from iliac crest RIA aspirates from the femur contained a significantly higher percentage of CD34 + progenitor cells, a significantly higher concentration of MSC and a significantly higher concentration of early EPC. The percentage of late EPC did not differ between both sites. Moreover, the capability of MSC for calcium deposition was significantly enhanced in MSC obtained with RIA [47]. In a subsequently following study, we hypothesized that the harvest procedure influences the osteogenic activity of human MSC rather than the tissue site itself. We generally were able to reproduce that concentration and osteogenic capacity of MSC harvested with RIA is higher compared to MSC from the iliac crest. We observed that the harvest procedure is a critical factor in osteogenesis of MSC in vitro. The altered gene expression and function of femur-derived MSC (RIA) might be due to the harsh isolation procedure [57].

### Scaffolds

Oftentimes, to spatially restrict regenerative cells, cells will be seeded on a carrier before being placed into the bone defect. Different kinds of scaffolds are available which vary in their chemical composition, shape and surface characteristics. Osteoconductivity, osteoinductivity and adherence of cells are dependent on material properties. A great variety of scaffolds belonging to different classes are commercially available. Those include synthetic scaffolds based on minerals present in bone such as hydroxyapatite or beta-tricalciumphosphate (β-TCP), other synthetic materials are based on derivates of polylactic acid. Non-synthetic scaffolds are frequently based on processed bovine cortical bone and spongiosa, scaffolds based on differentially processed bone obtained from human donors are also available.

Ideal biomaterials for bone reconstruction should fulfill requirements including mechanical stability, osteoinductivity, osteoconductivity and support of revascularization. It is generally accepted that the main aspects of the scaffold’s biological impact were pore size, certain surface micro- and nanostructure, stiffness and the release of putatively beneficial ions such as Ca^2+^. However, currently available single component materials do not meet all these requirements, despite increasing research efforts in this field. Hence, more sophisticated biomaterials are needed and combining different biodegradable biomaterials with complementary properties may circumvent individual shortcomings.

#### Assessment of scaffold cytocompatibility

Actually, there is a high demand for cytocompatibility testing, since the effect of the scaffold on cells is not predictable solely based on information about the scaffold’s chemical and physical properties. Therefore, we established a panel of assays that allows us to rate the cytocompatibility of a scaffold for BMC, EPC and MSC in a 96-well plate scale. Our test panel includes the assessment of seeding efficacy, metabolic activity, relative number of adhering cells, evaluation of functional aspects such as the secretion of VEGF and expression of genes relevant for vasculogenesis and osteogenesis.

We observed significant differences of cytocompatibility between different sorts of scaffolds, which were consistently found for each cell type that was analyzed. In particular, the relevance of the physical surface characteristics was demonstrated. It was observed that number, metabolic activity and gene expression of MSC, respectively, EPC, differed significantly when seeded on β-TCP scaffolds being chemically identic but different in their surface topography. Both cell types demonstrated a high adhesion and survival rate on the β-TCP offering a smooth surface, whereas cell number and cell activity rapidly declined on the rough material [58, 59].

**Fig. 3:**
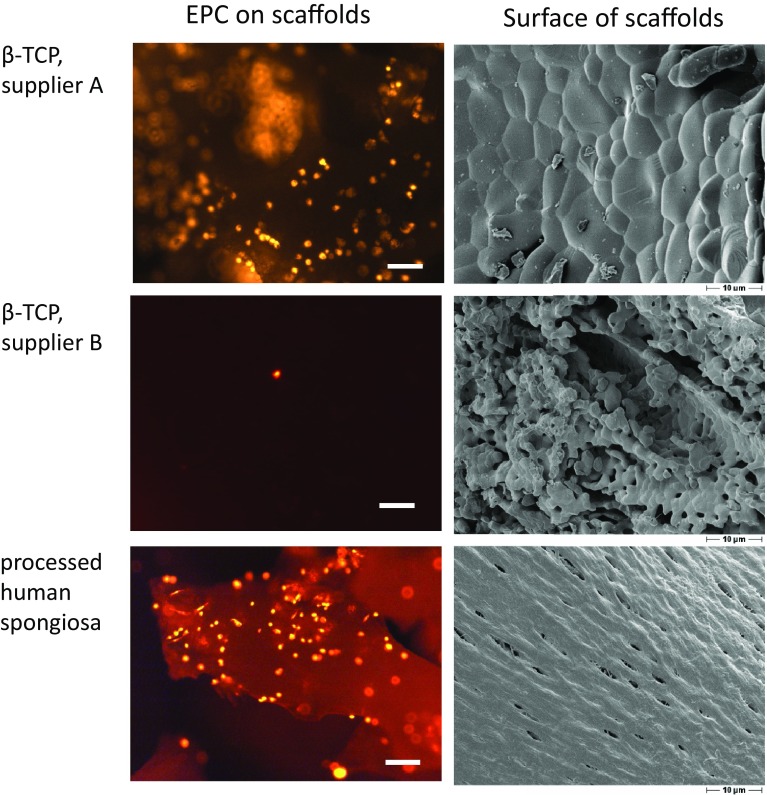
Differential adhesion of EPC on biomaterials. Adhesion of cells is strongly correlated to the surface characteristics of the biomaterial. Despite being chemically identic, cells show tremendously different adhesion on β-TCP from supplier A compared to the β-TCP from supplier B. Note the different surface structures of the materials. Natural materials based on processed human bone demonstrate generally a good cytocompatibility

Natural materials on the basis of human processed bone material demonstrated a high cytocompatibility for MSC, EPC [58–60] and BMC [49] that was superior to synthetic materials with regard to initial adherence and long-term survival (Fig. 3).

The importance of certain ions being released from the scaffold for the differentiation, survival and activity of EPC was demonstrated using a composite material developed in our department consisting of a PLA carrier combined with up to 40% bioglass (BG40, CaO-SiO_2_–SiO_2_ 80 mol-%, CaO 20 mol-%) [61]. BG40 released the most calcium, and improved endothelial differentiation and vitality of EPC best. This effect was mimicked by adding an equivalent amount of calcium to the medium and was diminished in the presence of the calcium chelator, EGTA.

### Experimental in vivo transfer studies

#### Transplantation of pre-cultivated progenitor cells improves bone healing in vivo

**Fig. 4:**
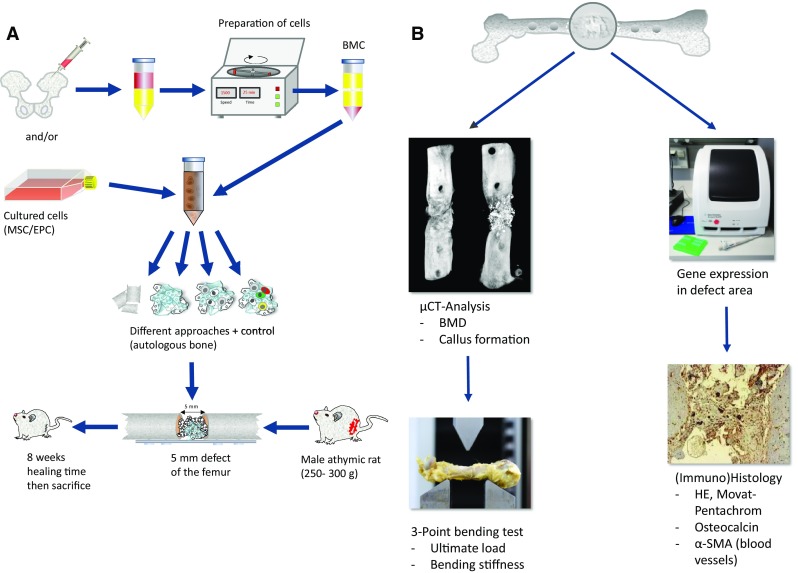
General scheme of the experimental setup to test various human cell types or scaffolds regarding their effect on bone healing is depicted in (**a**). The analyses made to evaluate the bone-healing response consist of µCT analysis to evaluate BMD and architecture of the new formed bone in the defect area, the same samples will be then used to determine the mechanical strength of the defect site using the three-point bending test. Additionally, RT-PCR to analyze the expression of genes involved in bone repair is performed using small samples from the defect site. Those bones were subsequently subjected to (immuno) histology to localize structures, cell types and protein expression in the bone defect (**b**)

We also analyzed the portability of the in vitro results to the in vivo situation. The general proceeding of those experiments is shown in Fig. 4.

Keeping our initial hypothesis in mind, that the combination of cells with complement properties is more effective for the bone defect healing compared to approaches using single cell sorts, we evaluated the effect of EPC alone or in combination with mesenchymal stem cells (MSC) on the early vascularization and bone healing in our critical size defect model of the athymic rat.

We were able to show that early vascularization after 1 week was significantly improved in the EPC/MSC group and the EPC group. The formation of a primitive vascular plexus was also detectable in the β-TCP, MSC, or autologous bone group, but on a significantly higher level, if EPC were transplanted alone or combined with MSC. The degree of early vascularization correlated well with the release of VEGF into the tissue, suggesting a paracrine effect of the transplanted EPC.

Concomitantly, bone defect healing after 8 weeks was most prominent, if MSC and EPC were transplanted into the bone defect compared to all other groups. Those findings indicated a synergistic effect between EPC and MSC and that the initial stage of neovascularization mediated by EPCs is crucial for complete bone healing in the late phase [62, 63].

The same positive effect of co-transplanted MSC and EPC on bone healing and vascularization was seen in a rat critically sized calvarian defect model using syngenic MSC and EPC seeded on a newly developed scaffold consisting of polylactic acid reinforced with 40% bioglass [61, 64].

#### BMC in bone healing: preclinical studies

Despite their beneficial effects on bone healing, the use of culture expanded cells comes with inherent disadvantages, including regulatory ones. To obtain a sufficient number of cells for clinical use, MSC will require several weeks of expansion in culture, markedly delaying definitive surgical repair of the bone defect. There is some evidence that, during that process, MSC may accumulate genetic alterations, which in turn might increase the risk of cancer [65]. Also, some of the growth factors that are used for EPC differentiation in vitro such as IGF-1 might support transformation of hematopoietic progenitors [66] from which EPC develop [51].

Bone marrow mononuclear cells (BMC) might be a promising alternative to cultured cells, if preliminary data about their osteoinductive properties can be confirmed in humans, specifically also in humans with pathological bone structure. Comparative data regarding the needs of BMC for the adhesion on biomaterials and biocompatibility to various biomaterials are lacking to a large extent. Therefore, we evaluated whether a surface coating would enhance human BMC adhesion and analyze the biocompatibility of three different kinds of biomaterials. β-TCP, demineralized bone matrix (DBM), and bovine cancellous bone (BS) were assessed. The seeding efficacy of BMC on uncoated biomaterials is generally high, although there are differences between these biomaterials. β-TCP and DBM were similar and both superior to BS. Those in vitro results could be generally confirmed using our femur defect model of the rat. Superior bone healing responses of the β-TCP and DBM scaffolds compared to BS were observed suggesting either as suitable materials for spatial restriction of BMC used for regenerative medicine purposes in vivo [49, 67]. Based on those preliminary data, we analyzed the impact of BMC seeded on a β-TCP scaffold in comparison with combined EPC and MSC using the same scaffold in our femur defect model of the male athymic rat in vivo. We observed less chondrocytes and a significantly more advanced bone formation in the BMC and EPC/MSC group in comparison with the control group (β-TCP without cells) after 8 weeks. Concomittantly, biomechanical stability of the defect area was significantly enhanced if BMC and EPC/MSC were implanted compared to control. The degree of new bone formation and biomechanical stability was similar between the BMC and the EPC/MSC group. Furthermore, no tumor formation was found either macroscopically or histologically after 26 weeks of BMC implantation [68].

#### BMC in clinical use, phase I and phase IIa clinical trials

Based on our promising preclinical results, we established a cell-based bone regeneration procedure applicable in the whole field of bone defects after trauma, tumors, joint arthroplasty and in osteoporotic defects. We hypothesized that transplantation of BMC + β-TCP into a bone defect should be safe, feasible and should promote bone formation and bony bridging of the defect resulting in improved clinical outcomes. The clinical problem of these studies is always that in substantial bone defects mostly the defects are very heterogeneous, and additional problems, such as soft tissue defects, or additional injuries exist. To allow for a rather standardized clinical defect situation, we have chosen the situation of a displaced proximal humerus fracture, thus a metaphyseal defect. Such an approach was proposed by Saxer et al. for their studies on adipose-derived stem cells [69].

Protocols for a German Medicines Law GCP trial were prepared and permissions from the local ethics board [No. 350/12] and the federal authority (PEI) [No. 1769] were obtained for treatment of 10 consecutive, eligible, consenting patients. We generated formal study protocols, including IMPD, and applied for § 40 AMG permission from the PEI for this phase I trial (EudraCT-Nr.:2012-004037-17, Date of registration: 30th of August 2012; Date enrolled first participant: 11th of September 2013). A manufacturing license for tissue procurement acc. to § 20b German Medicines Law and for manufacturing of the advanced therapy medicinal product (ATMP) “BMC2012” acc. to § 13 German Medicines Law, the autologous cell-based study drug, was obtained from the local regulatory agency (Regierungspräsidium Darmstadt). The study was registered in the European Clinical Trial Register as EudraCT No. 2012-004037-17.

After regulatory approval 10 patients were recruited after informed consent and completed follow-up between September 2013 and 2014 and published in 2016 [70].

Criteria for inclusion to this clinical trial were 2-, 3- or 4-fragment fracture (Neer classification), dislocation of ≥ 10 mm between fragments and/or angle of ≥ 45° between fragments and/or dislocation of tuberculum major of ≥ 5 mm, age > 18 years, informed consent for surgery and study participation.

The study was a single-arm uncontrolled study. All patients received cell-based therapy with autologous BMC: open reduction and internal fixation (ORIF) of the fracture, augmentation with composite of an acellular bone graft substitute (β-TCP) and BMC. Concentration of BMC was 1.3 × 10^6^ BMC/ml β-TCP analogous to the prior animal experiments.

Five follow-up visits for clinical and radiological control up to 12 weeks were performed and neither morbidity at the harvest site nor morbidity at the surgical wound site was observed. Furthermore, neither local nor systemic inflammation was noted. All fractures healed within the observation time without secondary dislocation. We conclude that cell therapy with autologous BMC for bone regeneration appeared to be safe and feasible with no drug-related adverse reactions being described to date. The impression of efficacy was given, although the study was not powered nor controlled to detect such [70].

**Fig. 5:**
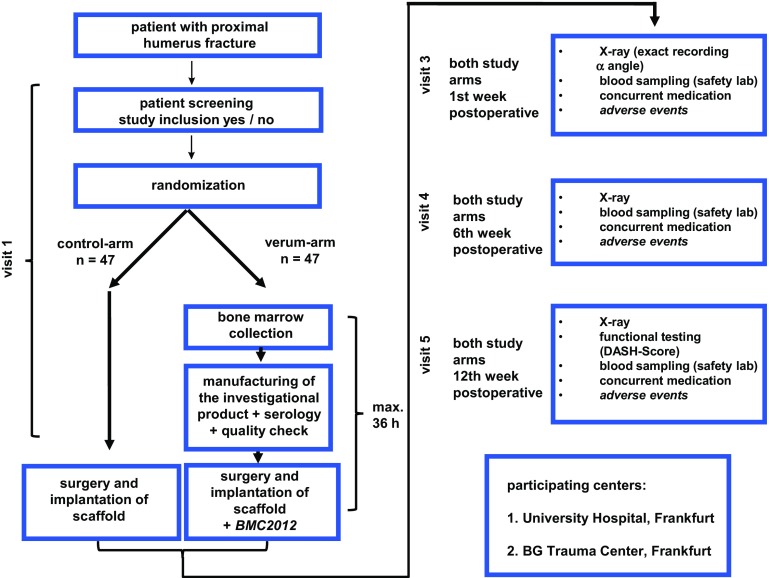
Study design of the BMC IIa-clinical trial (EudraCT-Nr.:2015-001820-51)

Therefore, a phase IIa-clinical trial was initiated to evaluate the effect of autologous BMC on bone healing. Formal study protocols for a multicentric, open, randomized phase IIa trial (EudraCT-Nr.:2015-001820-51) were generated and approval of the local ethics board [369/15] was obtained. A total of 94 patients distributed prospectively and randomly in a 1:1 relation to verum (BMC) or control group (β-TCP) is estimated and until June 2017, 24 patients have been already enrolled. We expect a study duration of 2–2.5 years and are eager to see if the phase I results can be demonstrated in a prospective randomized trial. The study design is shown in Fig. 5.

## Discussion

Alternative approaches ultimately based on the transplantation of vital bone-derived cells, respectively, bone material within the operative procedure by direct separation were previously evaluated by other groups [71, 72]. But up to date the large majority of patients still receive complete cancelleous bone graft from iliac crest or femur [73], which has the disadvantage of donor-site morbidity and limited material. Other approaches such as the use of nonviable scaffolds [74] cannot demonstrate a sufficient biological activity and guided bone healing. Thus, the advantages of using minimally manipulated cell drugs as opposed to ex vivo-cultivated stem cells are apparent. These include the risk of transmitting infectious agents with the cells, high laboratory costs and the risk, although probably small, of malignant transformation of long-term cultured cells [63–65].

Other treatment options to bone marrow processing for enrichment of vital progenitor cells have also been taken. Thus, concentrated autologous bone marrow aspirate was implanted together with a scaffold consisting of hydroxyapatite into bone defects and reportedly lead to a significant bone healing in almost all cases [75]. Of note, although clearly fulfilling the criteria of an advanced therapy medicinal product (ATMP) and hence requiring a manufacturing authorization and some kind of marketing authorization, these cell products were not regulator-approved at that time.

The metaphyseal fracture model we use in our clinical study was chosen to show a general effect of BMC in bone healing in human. The model is consistent feasible and comparable. In rats, we were able to show that BMC support bone healing in diaphyseal segmental defects. To the effectiveness of BMC in diaphyseal segmental defects in human further models are being developed.

## Conclusion and perspective

Our research history demonstrated the great potential of various stem cell species to support bone defect healing. It was clearly shown that the combination of different cell types is superior to approaches using single cell types. We further demonstrate that it is feasible to translate preclinically developed protocols from in vitro to in vivo experiments and follow positive convincing results into a clinical setting to use autologous stem cells to support bone healing.

With this review, we aimed to demonstrate a possible translational pathway from in vitro over experimental in vivo data to the clinical situation, which is possible in an academic setting. Furthermore, the clinical studies allow again a translation from the clinic to the bench. In particular, we attempt currently to improve the BMC-supported observed bone healing by further studies including optimization of scaffold formulations, evaluation of optimal cell concentrations, improvement of angiogenic and osteogenic properties of BMC by modification of certain µRNAs and of the analysis of effective cell populations within BMC. These experiments are performed in parallel with the phase IIa clinical study to improve hopefully possible further research.
